# Elevated Plasma Endothelin-1 and Pulmonary Arterial Pressure in Children Exposed to Air Pollution

**DOI:** 10.1289/ehp.9641

**Published:** 2007-04-27

**Authors:** Lilian Calderón-Garcidueñas, Renaud Vincent, Antonieta Mora-Tiscareño, Maricela Franco-Lira, Carlos Henríquez-Roldán, Gerardo Barragán-Mejía, Luis Garrido-García, Laura Camacho-Reyes, Gildardo Valencia-Salazar, Rogelio Paredes, Lina Romero, Hector Osnaya, Rafael Villarreal-Calderón, Ricardo Torres-Jardón, Milan J. Hazucha, William Reed

**Affiliations:** 1 Instituto Nacional de Pediatría, Mexico City, Mexico; 2 The Center for Structural and Functional Neurosciences, University of Montana, Missoula, Montanta, USA; 3 Inhalation Toxicology and Aerobiology Section, Safe Environments Programme, Health Canada, Ottawa, Ontario, Canada; 4 Escuela Médico Militar, Universidad del Ejército y Fuerza Aérea, México; 5 Departamento de Estadística, Universidad de Valparaíso, Valparaíso, Chile; 6 Centro de Ciencias de la Atmósfera, Universidad Nacional Autónoma de México, Mexico City, Mexico; 7 Department of Medicine; 8 Center for Environmental Medicine, Asthma and Lung Biology and; 9 Department of Pediatrics, University of North Carolina, Chapel Hill, North Carolina, USA

**Keywords:** air pollution, endothelial dysfunction, endothelin-1, children, particulate matter, pulmonary arterial pressure

## Abstract

**Background:**

Controlled exposures of animals and humans to particulate matter (PM) or ozone air pollution cause an increase in plasma levels of endothelin-1, a potent vasoconstrictor that regulates pulmonary arterial pressure.

**Objectives:**

The primary objective of this field study was to determine whether Mexico City children, who are chronically exposed to levels of PM and O_3_ that exceed the United States air quality standards, have elevated plasma endothelin-1 levels and pulmonary arterial pressures.

**Methods:**

We conducted a study of 81 children, 7.9 ± 1.3 years of age, lifelong residents of either northeast (*n* = 19) or southwest (*n* = 40) Mexico City or Polotitlán (*n* = 22), a control city with PM and O_3_ levels below the U.S. air quality standards. Clinical histories, physical examinations, and complete blood counts were done. Plasma endothelin-1 concentrations were determined by immunoassay, and pulmonary arterial pressures were measured by Doppler echocardiography.

**Results:**

Mexico City children had higher plasma endothelin-1 concentrations compared with controls (*p* < 0.001). Mean pulmonary arterial pressure was elevated in children from both northeast (*p* < 0.001) and southwest (*p* < 0.05) Mexico City compared with controls. Endothelin-1 levels in Mexico City children were positively correlated with daily outdoor hours (*p* = 0.012), and 7-day cumulative levels of PM air pollution < 2.5 μm in aerodynamic diameter (PM_2.5_) before endothelin-1 measurement (*p* = 0.03).

**Conclusions:**

Chronic exposure of children to PM_2.5_ is associated with increased levels of circulating endothelin-1 and elevated mean pulmonary arterial pressure.

Activation of the endothelin system and vasoconstriction has been reported in animal models (Bouthillier et al. 1998; Kang et al. 2002; Thomson et al. 2004, 2005; Vincent et al. 2001a) and humans (Brook et al. 2002; Calderón-Garcidueñas et al. 2005; Vincent et al. 2001b) after exposure to air pollutants. Endothelin-1 (ET-1) is implicated directly in the progression of cardiovascular diseases (Luscher and Barton 2000), and a number of polymorphisms for ET-1 and endothelin receptor genes have been identified that are associated with increased risk for pulmonary and cardiovascular conditions (Charron et al. 1999; Dong et al. 2004; Immervoll et al. 2001; Jin et al. 2003). ET-1 is atherogenic (Ihling et al. 2001; Lerman et al. 1995), and exposure to air pollutants has been shown to accelerate atherosclerosis in animals (Sun et al. 2005; Suwa et al. 2002) and humans (Künzli et al. 2005). Therefore, recurrent or sustained elevation of circulating ET-1 constitutes a plausible cause of some acute and chronic adverse health effects of air pollutants (Thomson et al. 2005).

The ambient atmosphere in Mexico City is characterized by elevated concentrations of ozone (especially in the southwest), and particulate matter (in the northeast), that frequently exceed United States air quality standards. Particulate matter (PM) is categorized by aerodynamic diameter into three classes: coarse PM 2.5–10 μm in aerodynamic diameter (PM_10_), fine PM 0.1–2.5 μm in aerodynamic diameter (PM_2.5_), and ultrafine PM < 0.1 μm in aerodynamic diameter. In addition to particulate matter and O_3_, aldehydes, volatile and nonmethane organic compounds, alkane hydrocarbons, and lipopolysaccharide (LPS) (15.3–20.6 ng/mg) are other typical contaminants of ambient Mexico City air (Bonner et al. 1998). Because of moderate climatic conditions, children in Mexico City engage in play and outdoor physical activities throughout the year in the late morning and afternoon when the diurnal pollutant levels are at their maximum (Villarreal-Calderón et al. 2002). Exposure to such contaminated air may pose a significant health risk for children.

Clinically healthy children residing in Mexico City exhibit pulmonary hyperinflation and interstitial markings on chest X rays (Calderón-Garcidueñas et al. 2006), and an imbalance of serum cytokines with significantly increased concentrations of interleukin (IL)-6 and IL-10 compared with children residing in areas with low levels of pollution (Calderón-Garcidueñas et al. 2003). In the study reported here, we investigated the plasma concentrations of ET-1 in Mexico City children and children from a control city with low levels of air pollutants. Because pediatric radiologists have noticed prominent pulmonary arteries in anterior–posterior chest X rays of Mexico City children, and given that ET-1 regulates pulmonary arterial pressure (PAP), our secondary objective was to determine whether these children have increased PAP. Our third objective was to determine whether plasma ET-1 levels in Mexico City children were correlated with pollutant exposure levels.

## Materials and Methods

### Study areas and pollutant exposure estimates

We selected two urban areas, Mexico City and Polotitlán, for this field study. Mexico City is located in a high mountain basin 2,250 m above sea level. The control city, Polotitlán, is located in the Mexico State 114 km northwest of Mexico City at 2,380 m above sea level. Mexico City residents are chronically exposed to concentrations of criteria air pollutants that exceed the United States standards, whereas air pollutant levels rarely exceed the standards in Polotitlán. Criteria air pollutants were monitored in Mexico City by the government atmospheric monitoring system at four stations: two in the southwest (Pedregal and Coyoacán) and two in the northeast (Xalostoc and San Agustín). Each child’s residence and school was within 5 miles of one of these monitoring stations. The pollutants that consistently exceeded their respective standard in the preceding 5 years were O_3,_ PM_10,_ and PM_2.5_. Thus, for these pollutants we estimated the cumulative exposure levels for each child for 1, 2, and 7 days before the measurement of plasma ET-1 levels. Pollutant concentrations between 0700 and 1900 hr, when the children were most active, were used for these estimates.

### Study population

We studied two cohorts of clinically healthy children, 6–13 years of age: The control cohort from Polotitlán (*n* = 22), and the exposed cohort from Mexico City (*n* = 59). Mexico City children came from two areas, the southwest (*n* = 40) and the northeast (*n* = 19), which have different air pollutant profiles (Raga et al. 2001). All included children were physically active and regular participants in a variety of outdoor physical activities. The information obtained from each child and/or parent (usually the mother) included age, place and length of residency, daily outdoor time, household cooking methods, parents’ occupational history, family history of atopic illnesses and respiratory disease, and personal history of otolaryngologic and respiratory symptoms. The study protocol was approved by the Human Studies Committee of the Institutional Review Board of the National Institute of Pediatrics, Mexico City.

### Study protocol

Recruitment (by word of mouth) was done between July 2003 and December 2004. The children of parents who volunteered their participation made at least four visits to the facility. The first visit was a screening visit. The study inclusion criteria were nonsmoking household and negative personal smoking history and environmental tobacco smoke exposure; lifelong residency in Mexico City or Polotitlán; residency within 5 miles of air pollutant monitoring stations; age 6–13 years; full-term birth; no known exposures to local sources of air pollutants (e.g., proximity to car-painting shops, gas stations, factories, solvents, carpenter’ shops, printing business); unremarkable clinical histories, including negative history of hospitalizations for respiratory illnesses, negative personal and family histories of atopic diseases, no lower respiratory illnesses, febrile episodes, or vaccinations in the previous 3 months; no indoor pets; and negative history of frequent travels outside Mexico City, or to a large city in the case of control children. Those who qualified for the study came for a second visit to give written consent from the children’s parents and oral consent from the children themselves. Once qualified, they were scheduled for subsequent visits, which included a physical by a pediatrician, fasting blood draw, and the Doppler echocardiogram exam.

### Plasma ET-1 levels and blood tests

Fasting peripheral blood samples were taken between 0700 and 0900 hr for complete blood count with differential and the preparation of plasma for determination of ET-1 levels. A QuantiGlo ELISA was used for the determination of ET-1 concentrations in accordance with the manufacturer’s instructions (R&D Systems, Inc., Minneapolis, MN, USA). The mean minimum detectable ET-1 concentration was 0.064 pg/mL.

### Doppler echocardiography

Cardiovascular function was assessed by two-dimensional (2D), M-mode, and Doppler echocardiography. Standard 2D echocardiographic examinations were performed with each child in the supine left position in accordance with recommendations of the American Society of Echocardiography (Schiller et al. 1989). The parents were instructed to avoid caffeine-containing beverages 24 hr before the children’s examinations. The echocardiographic analysis was performed with commercially available ultrasound systems (Sonos 2500; Hewlett–Packard Co./Agilent Technologies, Andover, MA, USA) equipped with 2.5- and 3.5-mHz transducers. Parasternal long- and short-axis views, as well as apical four- and two-chamber views, were used for evaluation of the functions of the ventricles and the heart valves. The protocol placed highest priority on systolic PAP, tricuspide, and right ventricle measurements. Systolic PAP encompasses the pulsatile component of arterial load, which includes the characteristics of right ventricular ejection and the proximal pulmonary arteries and wave reflections (Chemla et al. 2004). We calculated mean pulmonary arterial pressure (MPAP) from the systolic pressure using the formula from Chemla et al. (2004). The MPAP reflects the steady component of flow and the functional status of the distal pulmonary vasculature (Chemla et al. 2004). The complete protocol imaged the morphology of all four cardiac chambers and valves, and evaluated valve function and right ventricular outflow.

### Statistical analyses

The primary variables of interest were ET-1 concentrations and MPAP. We performed analysis of variance by a parametric one-way analysis of variance and the Newman-Keuls multiple comparison post test. We calculated correlations between variables using Pearson’s correlation. We considered a two-sided type I error rate of 0.05 to be significant when comparing differences between group means. Data are expressed as mean ± SE. All the statistical computations were performed with the use of Stata 8.3 software (StataCorp., College Station, TX, USA) or GraphPad Prism version 3.3 (GraphPad Software Inc., San Diego, CA, USA).

## Results

### Demographics and physical exams

All participant children were from middle-class families who lived in single-family houses. No occupational toxic exposures were reported by parents or close relatives. Children slept in bedrooms with no carpeting and had open windows for ventilation. All households had kitchens separated from the living and sleeping areas and used gas for cooking. A physical examination performed by the pediatrician showed that vital signs were unremarkable in all participant children. Children in this study had anthropometric values (weight and height) within normal limits for their age and sex. The demographic, clinical, and laboratory data for the three cohorts are summarized in Table 1.

### Plasma ET-1 levels and pulmonary arterial pressures

**Figure 1:**
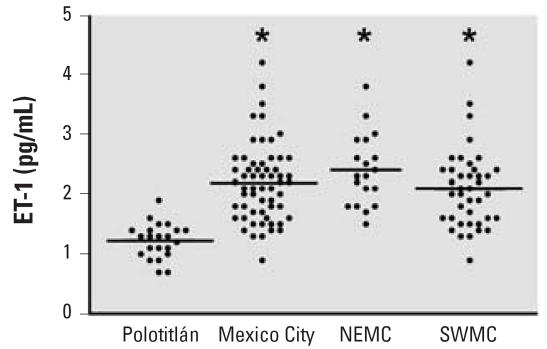
A scatterplot of plasma ET-1 levels by region. Mean plasma ET-1 levels for Mexico City children as a whole (*n* = 59), as well as for northeast (NEMC, *n* = 19) and southwest (SWMC, *n* = 40) Mexico City children analyzed separately were significantly greater than the mean for control (Polotitlán) children (*n* = 22). Horizontal bar indicates group means. *p < 0.001.

Compared with those from controls, mean plasma ET-1 concentrations were significantly higher in children from both northeast (*p* < 0.001; Figure 1, Table 1) and southwest (*p* < 0.001; Figure 1, Table 1) Mexico City as well as for all Mexico City children combined (2.24 ± 0.12 pg/mL; *p* < 0.001, Figure 1, Table 1). ET-1 levels tended to be higher in northeastern children than in southwestern children (Figure 1, Table 1), although the difference was not statistically significant.

**Figure 2:**
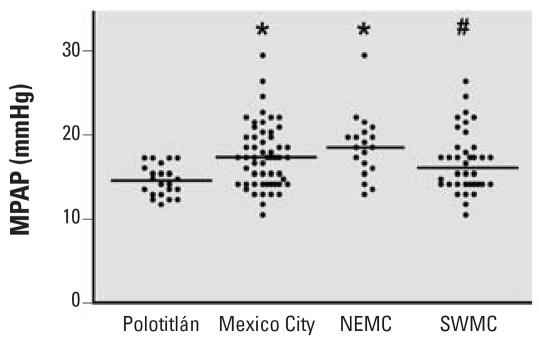
A scatterplot of MPAP by region. The average MPAP for Mexico City children as a whole (*n* = 59), as well as for northeast (NEMC, *n* = 19) and southwest (SWMC, *n* = 40) Mexico City children analyzed separately were significantly greater than the mean for control (Polotitlán) children (*n* = 22). Horizontal bar indicates group means. *p < 0.01; #*p* < 0.05.

**Figure 3:**
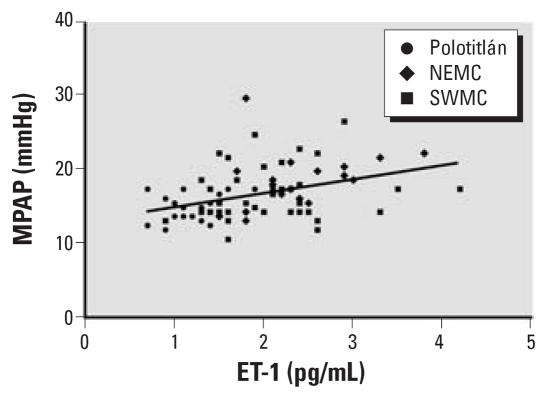
A plot of MPAP versus ET-1 levels for Polotitlán, northeast (NEMC), and southwest (SWMC) Mexico City children. MPAPs were significantly correlated with ET-1 levels (*r* = 0.43, *p* = 0.0001). A linear regression fit to the data is shown.

None of the children had cardiac anatomic abnormalities as assessed by echocardiography. Compared with the control cohort, the average MPAP, computed from systolic PAP determined by Doppler echocardiography, was significantly elevated in children from both northeast (*p* < 0.01; Figure 2, Table 1) and southwest (*p* < 0.05; Figure 2, Table 1) Mexico City, as well as for all Mexico City children combined (17.3 ± 0.5 mmHg, *p* < 0.01; Figure 2, Table 1). As was the case for ET-1 levels, MPAP tended to be higher in northeastern children than in southwestern children (Figure 2, Table 1). When children from all sites were considered, there was a significant positive correlation between MPAP and plasma ET-1 levels (*r* = 0.43, *p* < 0.0001, Figure 3).

Three children in the Mexico City cohort had MPAPs > 25 mmHg at rest and/or systolic pressure > 40 mmHg at rest, levels that are characteristic of pulmonary arterial hypertension (Simonneau et al. 2004). The three Mexico City children included two 8-year-old girls, one from the southwest and one from the northeast, and one 8-year-old boy from the southwest. All three had elevated plasma ET-1 (1.8, 1.9, and 2.9 pg/mL, respectively). Their systolic pressures ranged from 40 to 45 mmHg and their mean pressures ranged from 26 to 29 mmHg.

### White blood cell counts

**Figure 4:**
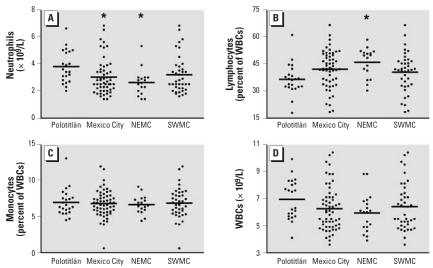
Scatterplots of complete blood count data by region. WBC, white blood cell. (*A*) Mean absolute neutrophil counts were significantly lower in Mexico City children (*n* = 56) than in control children (Polotitlán, *n* = 22). Northeast Mexico City children (NEMC, *n* = 17) had significantly lower neutrophil counts than control children (Polotitlán, *n* = 22). (*B*) The mean concentration of lymphocytes as a percentage of white blood cells was significantly higher in northeast Mexico City children (NEMC, *n* = 17) than in controls. (*C*) Monocyte concentrations were similar in northeast (NEMC, *n* = 17) and southwest (SWMC, *n* = 39) Mexico City and control (Polotitlán, *n* = 22) children. (*D*) White blood cell concentrations were not significantly different among Mexico City children (*n* = 59), Polotitlán children (*n* = 22), northeast (NEMC, *n* = 19) and southwest (SWMC, *n* = 40) Mexico City children. Horizontal bar indicates group means. *p < 0.05.

Children from northeast Mexico City exhibited significant decreases in both neutrophil absolute counts (*p* < 0.05) and neutrophils as a percentage of white blood cells (*p* < 0.01) when compared with controls (Figure 4A, Table 1). Children from southwest Mexico City had depressed neutrophil levels as well (Figure 4A, Table 1), but the differences between southwest Mexico City children and control children were not statistically significant. Even so, all Mexico City children taken together had statistically significant decreases in circulating neutrophil concentrations compared with controls (3.0 ± 0.2 × 10^3^/μL, *p* < 0.05; Figure 4A). The lowest absolute neutrophil counts recorded were 1.4 × 10^9^/L. No children had neutropenia defined as < 1 × 10^9^ neutrophils/L.

There was an increase in average lymphocyte concentrations in children from northeastern Mexico City (*p* < 0.05) and a smaller nonsignificant increase in lymphocyte concentrations in children from southwestern Mexico City (Figure 4B, Table 1) compared with control children. Average monocyte concentrations were essentially the same in Mexico City children (Figure 4C, Table 1) compared with controls. Average total white blood cell counts were lower in both northeastern and southwestern (Figure 4D, Table 1) Mexico City children, but the differences were not statistically significant.

### Correlation of ET-1 levels with outdoor hours and pollutant exposure

**Figure 5:**
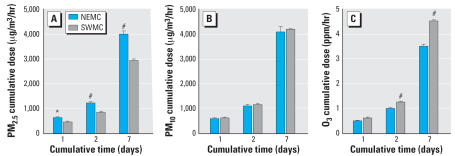
Estimated cumulative dose of PM_2.5_ (*A*), PM_10_ (*B*), and O_3_ (*C*) for northeast (NEMC) and southwest (SWMC) Mexico City averaged (± SE) over the 1-, 2-, and 7-day periods before measurement of ET-1 levels. The average dose calculations were based on estimates for each participating child. There were significant differences between the regions in the 1-, 2-, and 7-day cumulative PM_2.5_ dose and in the 2-day and 7-day cumulative O_3_ dose. *p < 0.05; #*p* < 0.001.

For Mexico City children, there was a significant, positive correlation between the number of hours spent outdoors every day (outdoor hours) and ET-1 levels (*r* = 0.31, *p* = 0.012). Likewise, there was a significant, positive correlation between outdoor hours and mean PAP (*r* = 0.42, *p* = 0.0008). This supported the notion that these effects are associated with exposure to air pollutants. To determine which air pollutants might be involved, we examined the acute cumulative exposure levels of PM_2.5_, PM_10_, and O_3_ for each Mexico City subject over 1, 2, and 7 days preceding the measurement of ET-1 levels. The average PM_2.5_ exposures for northeast Mexico City children over the 2- and 7-day cumulative periods preceding the measurement of ET-1 levels were significantly greater than for southwestern children (Figure 5A). In contrast, the average PM_10_ exposures were not significantly different for northeastern and southwestern children (Figure 5B). O_3_ exposures had a pattern that was the opposite of PM_2.5_ exposures. South-western children were exposed to significantly higher O_3_ levels than northeastern children over the 2-day and 7-day periods before ET-1 measurement (Figure 5C). Thus only PM_2.5_ exposures were greater in northeastern children than in southwestern children. This pattern was similar to the pattern of ET-1 levels, which tended to be higher in the northeast than in the southwest (Figure 1). When all Mexico City children were considered, there was a significant, positive correlation between ET-1 levels and the 7-day cumulative PM_2.5_ exposure (*r* = 0.28, *p* = 0.03).

## Discussion

Mexico City is located in a high mountain basin 2,250 m above sea level. Sunshine, light winds, temperature inversions, a basin setting, overcrowded population, heavy traffic, frequent urban leakage of liquefied petroleum gas, and intense industrial activity promote complex photochemical reactions producing a variety of oxidant chemicals and particulate matter. Because of the subtropical latitude and high altitude, the high concentrations of pollutants in Mexico City are seen throughout the year, with only small seasonal variation. Under these environmental conditions, children living in the city are most likely to be exposed to high doses of air pollutants. On school days, they spend significant amounts of time outdoors (3.94 ± 1 hr/day in this study), both during school exercise periods and after school (Villarreal-Calderón et al. 2002). On weekends the outdoor play time is even longer. This outdoor activity usually occurs during hours when air pollutant levels are near or exceed the standards. Healthy adult humans exposed to concentrated ambient PM_2.5_ and O_3_ experienced a significant brachial artery vasoconstriction (Brook et al. 2002), whereas exposure to PM_2.5_ alone elevated circulating ET-1 and ET-3 levels (Vincent et al. 2001b). Compared with adults, infants and children have much higher levels of plasma ET-1. Moreover, the number of ET-1 specific binding sites in infant’s and children’s hearts (both atria and ventricles) has been found to be significantly higher than in adults implying an enhanced physiologic function (Giannessi et al. 1999). Our data show that clinically healthy children living in Mexico City had increased concentrations of circulating ET-1 and MPAP and that ET-1 levels were positively correlated with daily outdoor hours (*p* = 0.012), and 7-day cumulative levels of PM_2.5_ (*p* = 0.03) before ET-1 measurement. These findings are consistent with controlled laboratory exposures of humans to air pollutants.

Several animal studies have reported increased levels of ET-1 after exposure to air pollutants (Bouthillier et al. 1998; Kang et al. 2002; Thomson et al. 2004, 2005; Vincent et al. 2001a). Inhaled O_3_ and urban particles have distinct toxicodynamics in rats with respect to regulation of lung preproET-1 and alteration of circulating ET-1 peptide levels. Whereas O_3_ causes a rapid response, detected immediately after exposure and subsiding within 24 hr, urban particles cause a more progressive and sustained response, with peak increase of plasma ET-1 24–36 hr after exposure (Thomson et al. 2005; Vincent et al. 2001a). The apparent predominant effect of PM_2.5_ on ET-1 in the present study is in keeping with these observations.

The lungs are the primary source of circulating endothelins, including ET-1. Mature endothelins have a half-life on the order of minutes due to rapid clearance from the bloodstream through binding to G-protein-coupled endothelin B (ET_B_) receptors in caveolae on the surface of lung capillary endothelial cells (Yamaguchi et al. 2003). Previous studies suggest that there are at least three mechanisms by which air pollutants could cause an increase in ET-1 levels. First, both O_3_ and PM can generate reactive oxygen species in tissues, a condition that has been linked to enhanced ET-1 expression (Kaehler et al. 2002). Second, ultrafine PM taken up by endothelial cell caveolae, as described in northeast Mexico City dogs (Calderón-Garcidueñas et al. 2001b), may directly interfere with binding of ET-1 to ET_B_ receptors, resulting in an increase in the half-life of circulating ET-1. Third, PM-associated LPS may increase preproET-1 mRNA transcription and stability. Douthwaite et al. (2003) exposed bovine aortic endothelial cells to LPS and found a concentration-dependent ET-1 release that was associated with increased transcription of preproET-1 mRNA and a 2-fold increase in preproET-1 mRNA half-life. This link between induction of ET-1 synthesis and LPS exposure ought to be considered in populations exposed to LPS, both in environmental and occupational settings. Environmental LPS is ubiquitous, so everyone is exposed to this biological pollutant. Mexico City has a variety of sources of environmental LPS (e.g., open field waste, waste disposal dust, wastewater treatment plants, open sewer channels, and daily outdoor deposits of thousands of pounds of animal and human fecal material) that contribute to measurable levels of LPS in Mexico City PM_10_ (Osornio-Vargas et al. 2003) and chronic exposure of Mexico City residents to LPS. Although the studies cited above present several plausible mechanisms by which chronic exposure to the complex mixture of air pollutants could induce sustained increases in plasma ET-1 concentrations, the extent of involvement of these mechanisms in humans remains to be determined.

The consequences of sustained elevations of ET-1 levels have been explored in animal models. Chronic expression of ET-1 in the lungs of ET-1 transgenic mice causes progressive pulmonary fibrosis and recruitment of inflammatory cells, predominantly CD4-positive cells (Hocher et al. 2000). Chronic perfusion of ET-1 in rats after 7 days increases pulmonary vascular resistance, an effect that disappears after 28 days of infusion possibly because of compensatory mechanisms (Migneault et al. 2005). In the same work, Migneault et al. (2005) demonstrated that chronic perfusion of ET-1 reduces the pulmonary vasodilator reserve in response to nitric oxide. The authors hypothesized that an ET-1–induced increase of reactive oxygen species production in both endothelial and smooth muscle cells contributes to a reduction in the bioavailability of nitric oxide (Migneault et al. 2005).

Acute exposure to air pollutants such as O_3_ and PM (especially fine and ultrafine PM) produces significant lung inflammation and injury that involves both epithelial and endothelial cells. Exposure to PM is also associated with a systemic inflammatory response that involves increased circulating levels of inflammatory mediators that can activate endothelium. Mexico City dogs exhibit focal peribronchiolar inflammatory infiltrates that surround the adjacent blood vessels, some of which contain platelet thrombi and marginated neutrophils (Calderón-Garcidueñas et al. 2001b). Moreover, pulmonary endothelial cells in these dogs contain free ultrafine PM in their cytoplasm, a situation that likely promotes the production of free radicals and endothelial damage (Calderón-Garcidueñas et al. 2001a). Children in Mexico City have fragmented red blood cells in peripheral blood smears, also suggestive of endothelial injury, most likely in the lung, because microthrombi are numerous in small vessels in the lungs of Mexico City dogs (Calderón-Garcidueñas et al. 2001a, 2001b). Moreover, ET-1 stimulates integrin-dependent adhesion of neutrophil granulocytes to endothelial cells (López et al. 1993), which could explain the decreases in the concentration and total number of circulating neutrophils in Mexico City children seen in this study. This notion is supported by analysis of lung tissue from healthy accidental-death victims from Mexico City showing neutrophils attached to damaged capillary endothelial cells (Calderón-Garcidueñas et al. 2007). Taken together, this evidence supports the notion that the systemic increase in ET-1 in Mexico City children could be a consequence of endothelial damage and dysfunction. Endothelial dysfunction is characterized by a shift in the actions of the endothelium toward reduced vasodilatation, a proinflammatory state, and prothrombic activities (Endemann and Schiffrin 2004). Endothelial dysfunction leads to chronic overproduction of vasoconstrictors such as ET-1 (Humbert et al. 2004).

It is noteworthy that all the children from northeast Mexico City in our study had plasma ET-1 levels that were above the control mean. Of 19 subjects from the northeast, 14 had ET-1 values higher than all controls. These data indicate that endothelial dysfunction and activation of the endothelin system in response to air pollutant exposure, given a sufficient PM dose, is a generalized effect rather than being restricted to a subset of sensitive individuals. This generalized effect for an environmental exposure to pollutants is in line with preliminary data from human subjects exposed to concentrated urban fine particles (Vincent et al. 2001b).

Mean pulmonary arterial pressures were elevated on average in Mexico City children, and the pressures correlated with ET-1 levels, as might be expected given the pulmonary vasoconstrictor effects of ET-1 and the repeated observations of increased circulating ET-1 in patients with elevated pulmonary arterial pressure (Fratz et al. 2003; Galie et al. 2004; Mathew et al. 2004). Three of the Mexico City children had MPAP levels at rest > 25 mmHg, a characteristic of pulmonary arterial hypertension (PAH). All children had a negative family history of PAH, no known risk factors for any disease that can cause PAH (Simonneau et al. 2004), and no other clinical symptoms of PAH. It is unclear at this point whether these and perhaps other Mexico City children will go on to develop PAH. Both adults and children living at high altitude, as all of our study subjects do, have a higher prevalence of elevated MPAP and are more prone to developing PAH. ET-1 overproduction is a plausible contributor to the pathogenesis of PAH (Galie et al. 2004), and pulmonary vasoconstriction is a likely early component of PAH pathogenesis that can be related to endothelial dysfunction (Humbert et al. 2004). Taken together, these observations warrant additional studies that follow Mexico City children for the development of clinical symptoms of PAH as they grow older.

A prooxidative, dysfunctional endothelium may contribute to a proatherogenic environment through an inappropriate regulation of vascular tone, permeability, coagulation, fibrinolysis, and cell adhesion and proliferation (Laight et al. 2000). Thus, endothelial dysfunction is recognised as an accessory in the pathogenesis of diabetic macroangiopathy, obesity, hypertension, dyslipidemia, and *in vivo* insulin resistance (Avogaro and De Kreutzenberg 2005; Laight et al. 2000). Dong et al. (2004) have identified at least one allele of the ET-1 gene (T1370G single nucleotide polymorphism) that confers an increased risk of left ventricular hypertrophy in response to environmental stress. Finally, ET-1 evokes cardiac mast cell degranulation (Murray et al. 2004), which can be arrhythmogenic. Indeed, extensive degranulation of mast cells is observed in healthy Mexico City dogs (Calderón-Garcidueñas et al. 2001a), and arrhythmias have been observed in Mexico City children (Calderon-Garcidueñas L, Hazucha MJ, Herbst MC, Reed W, Cascio WE, unpublished data). Taken together, these observations suggest that the elevated plasma ET-1 levels observed in this study may foreshadow the development of clinical cardio-pulmonary disease in Mexico City children.

## Conclusions

Chronic exposure of Mexico City children to a complex mixture of air pollutants was associated with a significant elevation of both plasma ET-1 concentration and MPAP. The prospective health effects of sustained elevations of plasma ET-1 and MPAP in growing children are unknown. It is plausible that a chronic exposure to significant levels of air pollutants, especially PM_2.5_, may lead to the development of clinically significant adverse health effects in a subpopulation of Mexico City children later in life. Our results clearly suggest a need for epidemiologic and toxicologic studies that can more fully characterize the association between sustained ET-1 and MPAP elevations and the development and progression of systemic health effects in this population.

## Figures and Tables

**Table 1:** Demographic, clinical, and laboratory data (mean ± SE) in the control (Polotitlán) and the northeast (NEMC) and southwest (SWMC) Mexico City cohorts.

Characteristic	Control	SWMC	NEMC
No.	22	40	19
Age (years)	7.4 ± 0.2	9.0 ± 0.3	7.2 ± 0.3
Sex (male/female)	9/13	20/21	9/10
Plasma ET-1 (pg/mL)	1.23 ± 0.06	2.40 ± 0.14	2.09 ± 0.10
Systolic pulmonary pressure (mmHg)	20.7 ± 0.7	24 ± 0.9	27.2 ± 1.4
Mean pulmonary pressure (mmHg)	14.6 ± 0.4	16.7 ± 0.6	18.6 ± 0.9
Fraction of children with pulmonary arterial pressure > 25 mmHg at rest	0/22	2/40	1/19
Sex (male/female)	0/0	1/1	0/1
Outdoor exposure time per day (hr)	4.3 ± 0.2	3.9 ± 0.2	4.0 ± 0.2
White blood cells (10^9^/L)	6.9 ± 0.3	6.4 ± 0.3	5.9 ± 0.3
Neutrophils (%)	54.1 ± 1.9	48.8 ± 1.8	44.4 ± 2.2
Neutrophils (10^9^/L)	3.8 ± 0.3	3.2 ± 0.2	2.6 ± 0.2
Lymphocytes (%)	36.4 ± 1.8	40.4 ± 1.7	45.8 ± 2.0
Monocytes (%)	6.9 ± 0.4	6.8 ± 0.3	6.6 ± 0.3
Platelets (10^9^/L)	312 ± 16	304 ± 11	299 ± 20
Hemoglobin (g/dL)	14.0 ± 0.1	14.2 ± 0.1	14.0 ± 0.1
Hematocrit (%)	41.1 ± 0.4	42.7 ± 0.3	41.5 ± 0.6

## References

[b1-ehp0115-001248] Avogaro A, De Kreutzenberg SV (2005). Mechanisms of endothelial dysfunction in obesity. Clin Chim Acta.

[b2-ehp0115-001248] Bonner JC, Rice AB, Lindroos PM, O’Brien PO, Dreher KL, Rosas I (1998). Induction of the lung myofibroblast PDGF receptor system by urban ambient particles from Mexico City. Am J Respir Cell Molec Biol.

[b3-ehp0115-001248] Bouthillier L, Vincent R, Goegan P, Adamson IY, Bjarnason S, Stewart M (1998). Acute effects of inhaled urban particles and ozone: lung morphology, macrophage activity, and plasma endothelin-1. Am J Physiol.

[b4-ehp0115-001248] Brook RD, Brook JR, Urch B, Vincent R, Rajagopalan S, Silverman F (2002). Inhalation of fine particulate air pollution and ozone causes acute arterial vasoconstriction in healthy adults. Circulation.

[b5-ehp0115-001248] Calderón-Garcidueñas L, Franco-Lira M, Torres-Jardón R, Henríquez-Roldán C, Barragán-Mejía G, Valencia-Salazar G (2007). Pediatric respiratory and systemic effects of chronic air pollution exposure: nose, lung, heart and brain pathology. Toxicol Pathol.

[b6-ehp0115-001248] Calderón-Garcidueñas L, Gambling TM, Acuña H, Garcia R, Osnaya N, Monroy S (2001a). Canines as sentinel species for assessing chronic exposures to air pollutants: part 2. Cardiac pathology. Toxicol Sci.

[b7-ehp0115-001248] Calderón-Garcidueñas L, Mora-Tiscareño A, Fordham LA, Chung CJ, Garcia R, Osnaya N (2001b). Canines as sentinel species for assessing chronic exposures to air pollutants: Part 1. Respiratory pathology. Toxicol Sci.

[b8-ehp0115-001248] Calderón-Garcidueñas L, Mora-Tiscareño A, Fordham LA, Chung CJ, Valencia-Salazar G, Gómez S (2006). Lung radiology and pulmonary function of children chronically exposed to air pollution. Environ Health Perspect.

[b9-ehp0115-001248] Calderón-Garcidueñas L, Mora-Tiscareño A, Fordham LA, Valencia-Salazar G, Chung CJ, Rodríguez-Alcaraz A (2003). Respiratory damage in children exposed to urban pollution. Pediatr Pulmonol.

[b10-ehp0115-001248] Calderón-Garcidueñas L, Romero L, Barragán G, Reed W (2005). Upregulation of plasma endothelin-1 (ET-1) levels and CD14 expression on peripheral blood monocytes in healthy children exposed to urban air pollution [Abstract]. FASEB J.

[b11-ehp0115-001248] Charron P, Tesson F, Poirier O, Nicaud V, Peuchmaurd M, Tiret L (1999). Identification of a genetic risk factor for idiopathic dilated cardiomyopathy. Involvement of a polymorphism in the endothelin receptor type A gene. CARDIGENE group. Eur Heart J.

[b12-ehp0115-001248] Chemla D, Castelain V, Humbert M, Hebert JL, Simonneau G, Lecarpentier Y (2004). New formula for predicting mean pulmonary artery pressure using systolic pulmonary artery pressure. Chest.

[b13-ehp0115-001248] Dong Y, Wang X, Zhu H, Treiber FA, Snieder H (2004). Endothelin-1 gene and progression of blood pressure and left ventricular mass: longitudinal findings in youth. Hypertension.

[b14-ehp0115-001248] Douthwaite JA, Lees DM, Corder R (2003). A role for increased mRNA stability in the induction of endothelin-1 synthesis by lipopolysaccharide. Biochem Pharmacol.

[b15-ehp0115-001248] Endemann DH, Schiffrin EL (2004). Endothelial dysfunction. J Am Soc Nephrol.

[b16-ehp0115-001248] Fratz S, Geiger R, Kresse H, Roemer G, Hennig M, Sebening W (2003). Pulmonary blood pressure, not flow, is associated with net endothelin-1 production in the lungs of patients with congenital heart disease and normal pulmonary vascular resistance. J Thorac Cardiovasc Surg.

[b17-ehp0115-001248] Galie N, Torbicki A, Barst R, Dartevelle P, Haworth S, Higenbottam T (2004). Guidelines on diagnosis and treatment of pulmonary arterial hypertension. The Task Force on Diagnosis and Treatment of Pulmonary Arterial Hypertension of the European Society of Cardiology. Eur Heart J.

[b18-ehp0115-001248] Giannessi D, Del Ry S, Andreassi MG, Nardini V, Pelosi G, Colombo MG (1999). High density of endothelin binding sites in the hearts of infants and children. Life Sci.

[b19-ehp0115-001248] Hocher B, Schwarz A, Fagan KA, Thone-Reineke C, El Hag K, Kusserow H (2000). Pulmonary fibrosis and chronic lung inflammation in ET-1 transgenic mice. Am J Respir Cell Molec Biol.

[b20-ehp0115-001248] Humbert M, Morrell NW, Archer SL, Stenmark KR, MacLean MR, Lang IM (2004). Cellular and molecular pathobiology of pulmonary arterial hypertension. J Am Coll Cardiol.

[b21-ehp0115-001248] Ihling C, Szombathy T, Bohrmann B, Brockhaus M, Schaefer HE, Loeffler BM (2001). Coexpression of endothelin-converting enzyme-1 and endothelin-1 in different stages of human atherosclerosis. Circulation.

[b22-ehp0115-001248] Immervoll T, Loesgen S, Dutsch G, Gohlke H, Herbon N, Klugbauer S (2001). Fine mapping and single nucleotide polymorphism association results of candidate genes for asthma and related phenotypes. Hum Mutat.

[b23-ehp0115-001248] Jin JJ, Nakura J, Wu Z, Yamamoto M, Abe M, Tabara Y (2003). Association of endothelin-1 gene variant with hypertension. Hypertension.

[b24-ehp0115-001248] Kaehler J, Sill B, Koester R, Mittmann C, Orzechowski HD, Muenzel T (2002). Endothelin-1 mRNA and protein in vascular wall cells is increased by reactive oxygen species. Clin Sci (Lond).

[b25-ehp0115-001248] Kang YJ, Li Y, Zhou Z, Roberts AM, Cai L, Myers SR (2002). Elevation of serum endothelins and cardiotoxicity induced by particulate matter (PM_2.5_) in rats with acute myocardial infarction. Cardiovasc Toxicol.

[b26-ehp0115-001248] Künzli N, Jerrett M, Mack WJ, Beckerman B, LaBree L, Gilliland F (2005). Ambient air pollution and atherosclerosis in Los Angeles. Environ Health Perspect.

[b27-ehp0115-001248] Laight DW, Carrier MJ, Anggard EE (2000). Antioxidants, diabetes and endothelial dysfunction. Cardiovasc Res.

[b28-ehp0115-001248] Lerman A, Holmes DR, Bell MR, Garratt KN, Nishimura RA, Burnett JC (1995). Endothelin in coronary endothelial dysfunction and early atherosclerosis in humans. Circulation.

[b29-ehp0115-001248] López FA, Riesco A, Espinosa G, Digiuni E, Cernadas MR, Alvarez V (1993). Effect of endothelin-1 on neutrophil adhesion to endothelial cells and perfused heart. Circulation.

[b30-ehp0115-001248] Luscher TF, Barton M (2000). Endothelins and endothelin receptor antagonists: therapeutic considerations for a novel class of cardiovascular drugs. Circulation.

[b31-ehp0115-001248] Mathew R, Huang J, Shah M, Patel K, Gewitz M, Sehgal PB (2004). Disruption of endothelial-cell caveolin-1alpha/raft scaffolding during development of monocrotaline-induced pulmonary hypertension. Circulation.

[b32-ehp0115-001248] Migneault A, Sauvageau S, Villeneuve L, Thorin E, Fournier A, Leblanc N (2005). Chronically elevated endothelin levels reduce pulmonary vascular reactivity to nitric oxide. Am J Respir Crit Care Med.

[b33-ehp0115-001248] Murray DB, Gardner JD, Brower GL, Janicki JS (2004). Endothelin-1 mediates cardiac mast cell degranulation, matrix metalloproteinase activation, and myocardial remodeling in rats. Am J Physiol Heart Circ Physiol.

[b34-ehp0115-001248] Osornio-Vargas AR, Bonner JC, Alfaro-Moreno E, Martínez L, García-Cuellar C, Ponce-de-León-Rosales S (2003). Proinflammatory and cytotoxic effects of Mexico City air pollution particulate matter *in vitro* are dependent on particle size and composition. Environ Health Perspect.

[b35-ehp0115-001248] Raga GB, Baumgardner D, Castro T, Martínez-Arroyo A, Navarro-González R (2001). Mexico City air quality: a qualitative review of gas and aerosol measurements (1960–2000). Atmos Environ.

[b36-ehp0115-001248] Schiller NB, Shah PM, Crawford M, DeMaria A, Devereux R, Feigenbaum H (1989). Recommendations for quantitation of the left ventricle by two-dimensional echocardiography. American Society of Echocardiography Committee on Standards, Subcommittee on Quantitation of Two-Dimensional Echocardiograms. J Am Soc Echocardiogr.

[b37-ehp0115-001248] Simonneau G, Galie N, Rubin LJ, Langleben D, Seeger W, Domenighetti G (2004). Clinical classification of pulmonary hypertension. J Am Coll Cardiol.

[b38-ehp0115-001248] Sun Q, Wang A, Jin X, Natanzon A, Duquaine D, Brook RD (2005). Long-term air pollution exposure and acceleration of atherosclerosis and vascular inflammation in an animal model. JAMA.

[b39-ehp0115-001248] Suwa T, Hogg JC, Quinlan KB, Ohgami A, Vincent R, van Eeden SF (2002). Particulate air pollution induces progression of atherosclerosis. J Am Coll Cardiol.

[b40-ehp0115-001248] Thomson E, Goegan P, Kumarathasan P, Vincent R (2004). Air pollutants increase gene expression of the vasoconstrictor endothelin-1 in the lungs. Biochim Biophys Acta.

[b41-ehp0115-001248] Thomson E, Kumarathasan P, Goegan P, Aubin RA, Vincent R (2005). Differential regulation of the lung endothelin system by urban particulate matter and ozone. Toxicol Sci.

[b42-ehp0115-001248] Villarreal-Calderón A, Acuña H, Villarreal-Calderón J, Garduno M, Henríquez-Roldán CF, Calderón-Garcidueñas L (2002). Assessment of physical education time and after-school outdoor time in elementary and middle school students in south Mexico City: the dilemma between physical fitness and the adverse health effects of outdoor pollutant exposure. Arch Environ Health.

[b43-ehp0115-001248] Vincent R, Kumarathasan P, Goegan P, Bjarnason SG, Guenette J, Berube D (2001a). Inhalation toxicology of urban ambient particulate matter: acute cardiovascular effects in rats. Res Rep Health Eff Inst.

[b44-ehp0115-001248] Vincent R, Kumarathasan P, Mukherjee B, Gravel C, Bjarnason S, Urch B (2001b). Exposure to urban particles (PM_2.5_) causes elevations of the plasma vasopeptides endothelin(ET)-1 and ET-3 in humans [Abstract]. Am J Respir Crit Care Med.

[b45-ehp0115-001248] Yamaguchi T, Murata Y, Fujiyoshi Y, Doi T (2003). Regulated interaction of endothelin B receptor with caveolin-1. Eur J Biochem.

